# Identification of actions to be taken by managers to facilitate the return to work of cancer survivors: Consensus between managers and cancer survivors

**DOI:** 10.1186/s12889-022-14271-w

**Published:** 2022-10-12

**Authors:** B. Porro, S. J. Tamminga, A. G.E.M. de Boer, A. Petit, Y. Roquelaure, M. A. Greidanus

**Affiliations:** 1 Univ. Angers, CHU Angers, Univ. Rennes, Inserm, EHESP, IRSET (Institut de Recherche en Santé, Environnement et Travail) – UMR_S 1085, SFR ICAT, F-49000 Angers, France; 2 Department of Public and Occupational Health, Amsterdam UMC location University of Amsterdam, Meibergdreef 9, Amsterdam, The Netherlands; 3 Amsterdam Public Health research institute, Societal Participation & Health, Amsterdam, The Netherlands

**Keywords:** Cancer survivors, Managers, Return to work, Expert consensus, TRIAGE method, Employer

## Abstract

**Background:**

Managers are considered to be main stakeholders in the return to work (RTW) of cancer survivors. However, the perspectives of cancer survivors and managers differ on what managerial actions should be taken during the RTW of cancer survivors. This difference might put effective collaboration and successful RTW at risk. Therefore, this study aims to reach consensus among managers and cancer survivors on the managerial actions to be taken during the four different RTW phases of cancer survivors (i.e., Disclosure, Treatment, RTW plan, Actual RTW).

**Methods:**

The Technique for Research of Information by Animation of a Group of Experts (TRIAGE) was implemented with managers and cancer survivors (hereafter referred to as “experts”). An initial list of 24 actions was derived from a previous study. Firstly, for each action, fifteen experts were asked to indicate individually how important this action is per RTW phase (Likert scale from 1 – “Not important at all” to 6 – “Very important”). Consensus was reached when ≥ 80% (i.e., ≥ twelve experts) of the experts rated that action ≥5. Secondly, for each phase of the RTW process, the 15 actions with the highest percentage were discussed with eight experts during the collective consultation, except for the actions that already reached consensus. After discussion, the experts voted whether each action was important (“yes” / “no”) and consensus required ≥ 87.5% (i.e., ≥ seven experts) of the experts to consider an action as important.

**Results:**

Twenty-five managerial actions were finally retained for at least one of the RTW phases, e.g., Disclosure: “respect privacy” and “radiate a positive attitude”, Treatment: “show appreciation” and “allow sufficient sick leave”, RTW Plan: “tailor” and “communicate”, and Actual RTW: “support practically” and “balance interest”.

**Conclusion:**

Cancer survivors and managers reached consensus on the importance of 25 managerial actions, distributed into each phase of the RTW process. These actions should be considered an interplay of managerial actions by different stakeholders on the part of the employer (e.g., direct supervisor, HR-manager), and should be a responsibility that is shared by these stakeholders. The collective implementation of these actions within the company will help cancer survivors feel fully supported.

**Supplementary Information:**

The online version contains supplementary material available at 10.1186/s12889-022-14271-w.

## Background

Over the past two decades, improvements in cancer diagnostics and medical treatments have led to more favorable survival rates and better health outcomes following a cancer diagnosis [1, 2]. However, cancer survivors do live with numerous after-effects that may impact their quality of life and, for those of working age, their likelihood of being able to work sustainably [3, 4]. Returning to work after cancer is considered a desirable outcome, both from an individual, as well as an organizational and societal point of view [5, 6]. For cancer survivors themselves, a successful return to work (RTW) is essential for regaining a sense of normalcy, improving self-esteem, providing financial security, and maintaining social relationships [6–9]. It is, however, a complex and multifactorial process requiring the participation of many stakeholders, including the cancer survivors themselves, healthcare professionals, family, and involving both the social and professional environment [10–12]. Supporting the RTW of a cancer survivor therefore requires action from each of these stakeholders. Specific interventions and tools need to be developed according to these stakeholders’ support requirements [10, 11, 13, 14].

Managers are considered to be one of the main stakeholders in cancer survivor RTW [10, 15]. The term “cancer survivors” refers to every person who has been diagnosed with cancer and is currently still alive. The term “manager” refers to the specific person who represents the organization that employs the cancer survivor and guides them during their sick leave and RTW; for example, the line manager, direct supervisor, Human Resources (HR) manager, case manager or employer. Managers are in a position to guide cancer survivors from the moment of diagnosis right up to sustainable RTW, for example by creating a supporting and stimulating work environment, and/or by communicating with the cancer survivor [10, 11, 16, 17]. Despite a willingness to be supportive, they also report a lack of knowledge and skills to support cancer survivors during their sick leave and RTW [16, 18].

An earlier Delphi study showed the most important actions to be taken by managers for successful guidance focused on RTW, according to cancer survivors and managers themselves [10]. Four RTW phases were considered separately in the concerning study: Phase 1 – Disclosure: the period between disclosure of the cancer survivor’s illness to the manager and the first treatment; Phase 2 – Treatment: the period during which the cancer survivor is on sick leave as a result of his/her treatment; Phase 3 – RTW plan: the period in which concrete planning of and preparation for the cancer survivor’s returning to work take place (the cancer survivor is still on sick leave during this phase); and Phase 4 – Actual RTW: the period after returning to work, up until six months after a stable work situation is reached (the term “stable” refers to unchanged working hours and position at work) [18]. For each of these phases, several actions were selected such as “providing emotional support”, “communicating”, “assessing work ability” and “showing appreciation” [10]. This previous study noticed significant differences in the selection of important actions between managers and cancer survivors [10]. For example, managers reached consensus on actions such as “communicate” and “treat normally” whereas cancer survivors reached consensus on “handle unpredictability” [10]. Such differences might put effective collaboration between both parties at risk [10], while it is precisely this collaboration that is recognized to be a pre-requisite for successful RTW [19, 20]. These two stakeholders have different but complementary experiences of the RTW process after cancer [10, 12, 18]. It is therefore necessary to identify managerial actions that satisfy both managers and cancer survivors to promote a shared representation of the cancer survivor RTW process.

Numerous studies have identified differences in the process of returning to work for cancer survivors, depending on the legal context of the country or the policy of the company [21–23]. While the authorities are encouraging the development of European projects to promote the RTW of cancer survivors [24], it is plausible that these differences also impact the managerial actions to be implemented. The identification of managerial actions should therefore be replicated, within several countries, to better understand and discuss the divergences and convergences between these countries.

Methodologically, the identification of managerial actions in the Greidanus et al. study was carried out using two separate Delphi consensus surveys for managers and cancer survivors [10]. However, reaching consensus between managers and cancer survivors requires that the opinions of all experts be confronted in the same methodological process. Furthermore, in the event of disagreement, the Delphi method does not encourage exchanges between experts [25], whereas it is useful to add a discussion phase into the method when divergences emerge [26, 27]. This discussion phase facilitates the experts’ decision-making through direct interactions that stimulate their thought process, confront their points of view, and draw out new practical information. In other words, the sharing of various experiences allows each expert included in the consensus to approach the question posed from a point of view other than their own personal experience [26, 27]. The Technique for Research of Information by Animation of a Group of Experts (TRIAGE) is a consensus method characterized as a dynamic decision-making technique based on the constructivist perspective and facilitates these types of exchanges insofar as it assumes that the consensus is constructed collectively [26, 27]. By applying this technique, the study aimed at reaching a consensus between managers and cancer survivors on the actions to be taken by managers during the different RTW phases of cancer survivors.

## Methods

### Design

**Fig. 1:**
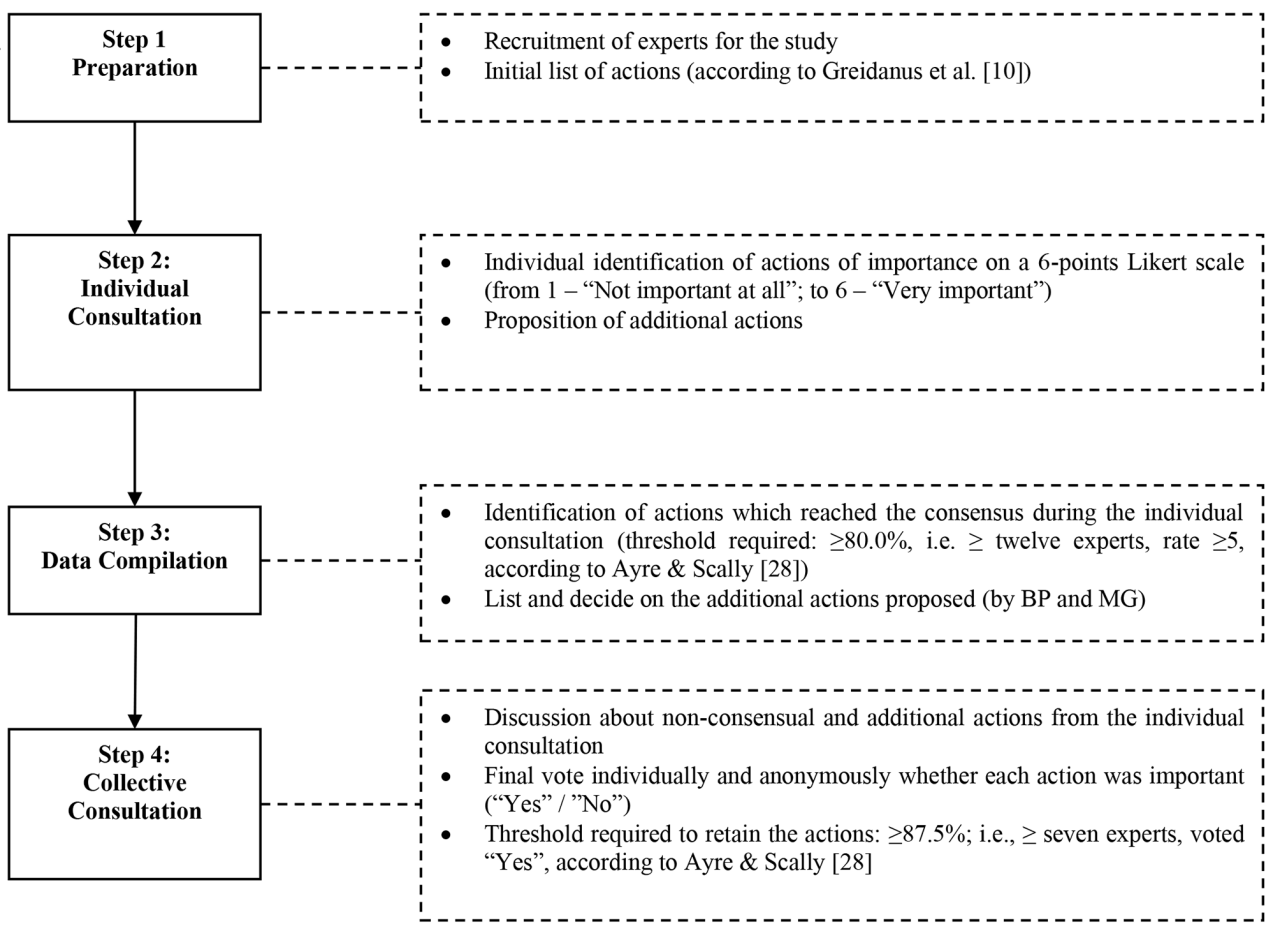
TRIAGE method procedure [22, 23]

TRIAGE can be described in four successive steps: (i) preparation; (ii) individual consultation; (iii) data compilation; (iv) collective consultation (Fig. 1) [26, 27]. Contrary to the initial method, the collective consultation step could not be conducted face-to-face due to the COVID-19 pandemic. In order to make it possible by videoconference, the data compilation and collective consultation steps were adapted as detailed below [11, 26, 27].

### Procedure

#### Preparation

The first preparatory step (March – June 2021) aimed to recruit experts for the study and to establish an initial list of managerial actions to be used for the individual consultation phase.

*Recruitment of experts.* In accordance with the guidelines of the TRIAGE method [10], six to twelve experts should be recruited for this study, comprising both cancer survivors and managers [10]. To be included in the study, cancer survivors must have been diagnosed with cancer within the last seven years, be employed at the time of diagnosis and have returned to work in France. Cancer survivors who were self-employed at the time of diagnosis or RTW were excluded. Managers must have supported at least one cancer survivor in their RTW in the last seven years. Different manager profiles were sought (i.e., first-line manager, HR manager, HR director, case manager, company director) to obtain a variety of perspectives according to the position within the companies. Particular attention was paid to each expert’s professional structure. Because of the diversity of profiles, it was not possible to keep to the maximum of twelve experts. Care was therefore taken to strike a balance between the advisable number of experts in order to adhere to the methodology of the study [26, 27], and the number of profiles required for the relevance of the study [10]. Managers were recruited through the business network of the Ligue Contre le Cancer (departmental committee of Loire Atlantique) [28] and the ReWork-QoL research program of the SIRIC ILIAD (Nantes & Angers) [29]. Companies from the business network were asked to relay the information to managers with the expected profile (see inclusion criteria). Cancer survivors have all been trained as “expert-patients” (i.e., experienced patients who have been trained in active listening, and who support other cancer survivors) [30], and were recruited through the Ligue Contre le Cancer (departmental committee of Loire Atlantique) and the AF3M [31], an association of patients diagnosed with multiple myeloma, a partner of the ReWork-QoL research program of the SIRIC ILIAD (Nantes & Angers) [29]. We asked our partner (the Ligue Contre le Cancer, and the AF3M) to relay the information to expert-patients with the expected profile (see inclusion criteria). When interested, potential participants were asked to contact the researcher in charge of the study (PB) to verify their eligibility for the study and to obtain information on what was expected from participation. Care was taken, when recruiting managers and cancer survivors, that none of the participating managers, supervised the work of any other expert.

*Initial list of actions.* To elaborate on previous work, an initial list of 24 managerial actions was derived from a previous study [10]. In turn, this list was based on a systematic review that identified employer-related barriers and facilitators for cancer survivor work participation [10, 16].

#### Individual consultation

The individual consultation (June 2021) aimed to assess each action included in the initial list of 24 actions based on their perceived importance by the experts, and to identify any additional actions. Each expert responded individually to an online questionnaire, using LimeSurvey software [32]. Firstly, the experts’ demographic and work-related characteristics were asked. Thereafter, experts were asked to indicate how important each action is “for a manager supporting the RTW of a cancer survivor employed by them” on each of the four RTW phases (i.e., phase 1 – Disclosure; phase 2 – Treatment; phase 3 – RTW plan; and phase 4 – Actual RTW), accompanied by the description of the phases as mentioned in the introduction [10, 18]. For this, a six-point Likert scale was used (from 1 = Not important at all; to 6 = Very important). Lastly, experts also had the opportunity to propose up to three additional actions per RTW phase.

#### Data compilation

The data compilation (July 2021) aimed to: (i) identify the actions on which consensus was reached during the individual consultation; and (ii) list and decide on the additional actions proposed by the experts. Consensus was reached when at least a certain percentage of the expert group, depending on the group size and in accordance with Ayre & Scally [33], selected the action as (very) important (i.e., scores “5” or “6”). In accordance with Greidanus et al. [10], for each phase of the RTW process, the 15 actions with the highest percentage (or more in case of a shared 15th place) were retained for the collective consultation. Of these 15 actions, the ones that reached the consensus threshold were automatically retained in the final list and were not included for the collective consultation, as the experts already reached consensus on those actions.

Additional actions were added to the collective consultation when: the action was indeed an action to be performed by the manager (i.e., a managerial action); and the action was not already covered by any of the other actions. Two authors (BP and MG) decided whether to add the additional actions to the collective consultation and all authors were consulted in case of disagreement.

#### Collective consultation

The collective consultation (September 2021) aimed to collectively discuss the proposed actions and come to an agreement about the non-consensual and additional actions from the individual consultation. This step was conducted by a trained moderator (BP) and two assistants (YR and AP). Experts had to discuss and then decide whether the managerial actions were of importance “for a manager supporting the RTW of a cancer survivor employed by them”. The collective consultation was conducted by videoconference that was audio- and videotaped with the agreement of all experts. All RTW phases and actions were addressed in turn. Each expert was asked to put forward one line of reasoning as to whether they believed the specific action to be of importance. All arguments, both for and against the specific action, were digitally noted by the moderator on a sheet that was visible to all attendees via the researcher’s shared screen [11]. Once all opinions were collected for the action, the experts were allowed to discuss the importance of this action for several minutes. The moderator ensured that everyone participated. Thereafter, experts used LimeSurvey to vote individually and anonymously on whether they thought the action was of importance (i.e., “Yes” or “No”). Consensus was reached when at least a certain percentage of the expert group, again depending on the group size and in according with Ayre & Scally [33], selected “Yes”. These actions were retained in the final list. Actions that did not reach the “Yes” consensus were excluded. The moderator provided feedback to the group immediately after voting.

## Results

### Recruitment of experts

Nine managers and ten cancer survivors were approached to participate in the study. One manager and one cancer survivor did not meet the inclusion criteria; two cancer survivors refused to participate due to lack of time. In the end, eight managers and seven cancer survivors were included (Table 1). The inclusion rate was 89% for managers, 70% for cancer survivors and 79% for all experts.

**Table 1:** Expert group characteristics

Managers (n = 8)				
			**Mean (SD)**	**n**
**Gender: Female**				5
**Age (years)**			49 (9)	
**Level of education**				
	Bachelor or less			4
	Bachelor of Honors			2
	Masters			2
**Company size (n employees)**				
	51–250			1
	251 or more			7
**Seniority in the company (years)**			20 (7)	
**Position in the company**				
	HR Director			2
	HR Manager			1
	Manager			3
	Case manager			1
	Young retired Head (less than three months)			1
**Seniority – position (years)**			15 (5)	
**Number of cancer survivors supported**				
	1			3
	2			1
	4			1
	5			1
	10 or more			2
**Time since last cancer survivor**				
	1 year or less			6
	2 years			1
	6 years			1
**Cancer survivors (n = 7)**				
			**Mean (SD)**	**n**
**Gender: Female**				6
**Age (years)**			56 (7)	
**Level of education**				
	Bachelor or less			2
	Bachelor of Honors			1
	Masters			4
**Cancer site**				
	Multiple myeloma			1
	Breast			4
	Colorectal			2
**Years since diagnosis**			6 (1)	
**Total duration of sick leave due to cancer (months)**			14 (15)	
**Company size (n employees)**				
	50 or less			2
	51–250			1
	251 or more			4
**Seniority in the company (years)**			15 (9)	
**Management position: yes**				5
**Current professional situation**				
	Working at the same company and in the same position			2
	Working at the same company, but in a different position			1
	Working at another company			2
	Early retirement			1
	Self-employed ^a^			1

*Notes.*^a^ Consistent with the inclusion criteria, the cancer survivor who is currently self-employed was an employee at the time of diagnosis and experienced the RTW process as an employee before becoming self-employed.

### Individual consultation

#### Initial list of actions

Fifteen experts participated in the individual consultation phase. According to Ayre & Scally, the threshold to reach consensus during the individual consultation phase was therefore 80.0% (i.e., ≥ twelve experts) [33]. Eighteen actions reached the 80.0% threshold of importance for at least one phase of the RTW process in the individual consultation as follows: four actions for phase 1 – Disclosure (i.e., “show appreciation”, “radiate a positive attitude”, “respect privacy” and “seek balance between privacy and support”); seven actions for phase 2 – Treatment (i.e., “show appreciation”, “communicate”, “allow sufficient sick leave”, “handle unpredictability”, “respect privacy”, “balance interests” and “seek balance between privacy and support”); 15 actions for phase 3 – Return to work plan (i.e., “support practically”, “assess work ability”, “show appreciation”, “communicate”, “support emotionally”, “allow sufficient sick leave”, “plan return to work”, “handle unpredictability”, “radiate a positive attitude”, “respect privacy”, “deal with colleagues”, “offer reintegration programs”, “balance interests”, “provide time for reorientation and retraining” and “seek balance between privacy and support”); and nine actions for phase 4 – Actual return to work (i.e., “support practically”, “assess work ability”, “show appreciation”, “communicate”, “adjust expectations”, “reduce work pressure”, “create a positive work atmosphere”, “balance interests” and “seek balance between privacy and support”) (Table 2). Furthermore, 20 actions needed to be discussed for at least one phase of the RTW process during the collective consultation as follows: twelve actions for phase 1 – Disclosure, nine for phase 2 – Treatment, none for phase 3 – Return to work plan, and nine for phase 4 – Actual return to work.

**Table 2:** Number of experts who considered the action important in the individual consultation phase (N = 15)

Actions	Phase 1: Disclosure n	Phase 2: Treatment n	Phase 3: Return to work plan n	Phase 4: Actual return to work n
1. **Support practically** – Provide the employee with cancer with practical support (e.g., adapting tasks, workplace and working hours)	*10*	4	**12**	**15**
2. **Assess work ability** – Assess the extent to which the employee with cancer is able to work in the right manner	*9*	2	**13**	**13**
3. **Show appreciation** – Give the employee with cancer the feeling that you want them back at work	**13**	**15**	**14**	**12**
4. **Communicate** – Communicate effectively with the employee with cancer (in terms of tone, intensity, subjects and channels)	*11*	**13**	**13**	**13**
5. **Support emotionally** – Support the employee with cancer emotionally (e.g., showing interest, being involved and understanding)	*11*	*9*	**12**	*9*
6. **Adjust expectations** – Adjust expectations regarding the performance of employee with cancer to their current situation	*9*	6	11	**12**
7. **Allow sufficient sick leave** – Allow sufficient sick leave and not putting pressure on the employee with cancer to return to work	*11*	**15**	**14**	8
8. **Treat normally** – Treat the employee with cancer as if they are not ill (e.g., avoid inappropriate treatment, including being too protective or concerned)	*11*	4	9	*9*
9. **Plan return to work** – Make a plan for the employee’s return to work in consultation with them	5	*9*	**13**	*10*
10. **Handle unpredictability** – Try to cope as well as possible with the unpredictability of the illness and the absence of the employee with cancer	*9*	**12**	**12**	*10*
11. **Reduce work pressure** – Reduce the pressure of work on the employee with cancer	*9*	*10*	11	**12**
12. **Radiate a positive attitude** – Radiate a positive attitude when guiding the employee with cancer	**13**	*11*	**13**	*11*
13. **Respect privacy** – Respect the privacy of the employee with cancer	**13**	**14**	**13**	*11*
14. **Deal with colleagues** – Inform and supervise colleagues of the employee with cancer	7	*9*	**12**	*9*
15. **Collaborate** – Collaborate with the employee with cancer	5	1	3	*9*
16. **Create a positive work atmosphere** – Create a positive atmosphere at work, whether or not the employee with cancer is present	*11*	*10*	11	**12**
17. **Offer reintegration programs** – Offer the employee with cancer external reintegration programs (e.g., third-party support services of fitness programs)	3	*9*	**12**	6
18. **Balance interests** – Try to cope as well as possible with the different interests at stake (e.g., those of the company, the employee with cancer and their colleagues)	*11*	**12**	**13**	**15**
19. **Provide time for reorientation and retraining** – Provide the employee with cancer with time for reorientation and retraining	5	5	**13**	*10*
20. **Seek balance between privacy and support** – Seek the right balance between respecting the privacy of the employee with cancer and offering them support	**12**	**12**	**13**	**12**
21. **Support financially** – Support the employee with cancer financially (e.g., continue to pay them during sick leave or help them with benefits applications)	*9*	*10*	8	5
22. **Comply with legislation** – Comply strictly with the obligations imposed by the law	5	6	7	6
23. **Search for external support for yourself (manager)** – Seek out external support for yourself as the employer of an employee with cancer (e.g., from the occupational physician, other employers or a psychologist). *Note that this external support does not target the employee.*	8	*9*	11	7
24. **Possess or seek knowledge of cancer** – Possess or seek out general knowledge of cancer, its treatment and its possible consequences for work	6	7	8	7

*Notes.* The numbers presented correspond to the number of experts who responded “5” or “6” on the Likert scale (from 1 - Not important at all; to 6 - Very important) for each action in each phase

**In bold**: actions on which consensus have been reached

*In italics*: actions to be discussed in the collective consultation

#### Additional actions

Eighty-eight additional actions were proposed by the experts across the four RTW phases: 24 actions for phase 1 – Disclosure, 22 actions for phase 2 – Treatment, 21 actions for phase 3 – Return to work plan, and 21 actions for phase 4 – Actual return to work. Sixty-seven additional actions were already covered by another managerial action, such as: “*Be flexible in working hours*” which was covered by the managerial action “support practically”; and “*Try to communicate regularly with the team about the employee’s health status, only if allowed”* which was covered by the managerial action “deal with colleagues”.

Twenty-one additional proposed actions were discussed by the research team, and collectively emerged in three additional actions: (i) “Listen: Listen actively to the difficulties and needs expressed by the employee with cancer” – covering, for example: “*Listen to what the employee needs: time, pace, mobility, responsibilities, accommodation, rather than proposing.*”; (ii) “Refer to internal reintegration programs: Refer to relevant experts within the company (e.g., occupational physician, human resources services, tutors, experiences experts)” – covering for example: “*Look for someone in the team with the necessary qualities to be a tutor, who can support him/her in certain tasks*”; and (iii) “Tailor: Tailor the support to the needs of the employee with cancer” – covering for example: “*Adapting to each case*”. These three actions were added for collective consultation on all four RTW phases.

### Collective consultation

Four cancer survivors did not participate in the collective consultation: one due to a recurrence of her cancer; two due to technical problems, and one for unclear reasons. In addition, three managers did not participate: two due to professional constraints, and one for unclear reasons.

Eight experts participated in the collective consultation, which lasted five hours. According to Ayre & Scally, the threshold to reach consensus during the collective consultation phase was therefore 87.5% (i.e., ≥ seven experts) [33]. The sample included three female cancer survivors (two breast cancer and one colorectal cancer), of which two were in management positions at the time of cancer diagnosis; and five managers (two males) working in a variety of managerial positions.

Of the actions discussed during the collective consultation, the experts reached consensus on the importance of the following: (i) eleven actions for phase 1 – Disclosure (i.e., “Communicate”, “Support emotionally”, “Allow sufficient sick leave”, “Treat normally”, “Handle unpredictability”, “Create a positive work atmosphere”, “Balance interests”, “Support financially”, “Listening”, “Refer to internal reintegration programs”, and “Tailoring”); (ii) nine actions for phase 2 – Treatment (i.e., “Support emotionally”, “Radiate a positive attitude”, “Deal with colleagues”, “Create a positive atmosphere”, “Support financially”, “Search for external support for yourself”, “Listening”, “Refer to internal reintegration programs”, and “Tailoring”); (iii) three for phase 3 – Return to work plan (i.e., “Listening”, “Refer to internal reintegration programs”, and “Tailoring”); and (iv) eleven for phase 4 – Actual return to work (i.e., “Support emotionally”, “Treat normally”, “Plan return to work”, “Handle unpredictability”, “Radiate a positive attitude”, “Respect privacy”, “Deal with colleagues”, “Collaborate”, “Listening”, “Refer to internal reintegration programs”, and “Tailoring”). The results of the collective consultation are available in Table S1 (Supplementary materials) and the final consensus results can be found in Table 3.

**Table 3:** Final actions on which the experts reached consensus

Actions	Phase 1: Disclosure	Phase 2: Treatment	Phase 3: Return to work plan	Phase 4: Actual return to work
1. **Support practically** – Provide the employee with cancer with practical support (e.g., adapting tasks, workplace and working hours)			X	X
2. **Assess work ability** – Assess the extent to which the employee with cancer is able to work in the right manner			X	X
3. **Show appreciation** – Give the employee with cancer the feeling that you want them back at work	X	X	X	X
4. **Communicate** – Communicate effectively with the employee with cancer (in terms of tone, intensity, subjects and channels)	X	X	X	X
5. **Support emotionally** – Support the employee with cancer emotionally (e.g., showing interest, being involved and understanding)	X	X	X	X
6. **Adjust expectations** – Adjust expectations regarding the performance of employee with cancer to their current situation				X
7. **Allow sufficient sick leave** – Allow sufficient sick leave and not putting pressure on the employee with cancer to return to work	X	X	X	
8. **Treat normally** – Treat the employee with cancer as if they are not ill (e.g., avoid inappropriate treatment, including being too protective or concerned)	X			X
9. **Plan return to work** – Make a plan for the employee’s return to work in consultation with them			X	X
10. **Handle unpredictability** – Try to cope as well as possible with the unpredictability of the illness and the absence of the employee with cancer	X	X	X	X
11. **Reduce work pressure** – Reduce the pressure of work on the employee with cancer				X
12. **Radiate a positive attitude** – Radiate a positive attitude when guiding the employee with cancer	X	X	X	X
13. **Respect privacy** – Respect the privacy of the employee with cancer	X	X	X	X
14. **Deal with colleagues** – Inform and supervise colleagues of the employee with cancer		X	X	X
15. **Collaborate** – Collaborate with the employee with cancer				X
16. **Create a positive work atmosphere** – Create a positive atmosphere at work, whether or not the employee with cancer is present	X	X		X
17. **Offer reintegration programs** – Offer the employee with cancer external reintegration programs (e.g., third-party support services of fitness programs)			X	
18. **Balance interests** – Try to cope as well as possible with the different interests at stake (e.g., those of the company, the employee with cancer and their colleagues)	X	X	X	X
19. **Provide time for reorientation and retraining** – Provide the employee with cancer with time for reorientation and retraining			X	
20. **Seek balance between privacy and support** – Seek the right balance between respecting the privacy of the employee with cancer and offering them support	X	X	X	X
21. **Support financially** – Support the employee with cancer financially (e.g., continue to pay them during sick leave or help them with benefits applications)	X	X		
22. **Comply with legislation** – Comply strictly with the obligations imposed by the law				
23. **Search for external support for yourself (manager)** – Seek out external support for yourself as the employer of an employee with cancer (e.g., from the occupational physician, other employers or a psychologist). *Note that this external support does not target the employee.*		X		
24. **Possess or seek knowledge of cancer** – Possess or seek out general knowledge of cancer, its treatment and its possible consequences for work				
25. **Additional action 1 – Listen**: Listen actively to the difficulties and needs expressed by the employee with cancer.	X	X	X	X
26. **Additional action 2 – Refer to internal reintegration programs**: Refer to the relevant experts within the company (e.g., occupational physician, social worker, human resources services, tutors, experience experts)	X	X	X	X
27. **Additional action 3 – Tailor**: Tailor the support to the needs of the employee with cancer	X	X	X	X

## Discussion

This study aimed to reach consensus among managers and cancer survivors on the actions to be taken by managers during the four RTW phases of a cancer survivor (Disclosure, Treatment, RTW Plan, Actual RTW). The experts reached consensus on the importance of 25 managerial actions, which were all important during at least one of the RTW phases (e.g., Disclosure – “Respect privacy”; Treatment – “Communicate”; RTW Plan – “Tailor”; Actual RTW – “Collaborate”). The following eleven managerial actions were deemed important throughout all RTW phases: “show appreciation”, “communicate”, “support emotionally”, “handle unpredictability”, “radiate a positive attitude”, “respect privacy”, “balance interest”, “seek balance between privacy and support”, “listen”, “refer to internal reintegration programs”, and “tailor”.

Managers’ professional support for the cancer survivors is essential in their RTW process [16]. A study by Nilsson et al. [34] showed that cancer survivors who perceived low levels of support from their manager were more likely to have longer sick leaves. Another study highlighted that this lack of support was not the result of a lack of willingness on the part of the manager but rather a lack of knowledge about how to provide this support [16]. Furthermore, a study by Yagil et al. [35], in which twelve dyads of cancer survivors with their supervisors were studied, pointed out that this support should be approach as teamwork. Finally, in a systematic review of qualitative studies on the RTW experiences of cancer survivors, many employer-related barriers and facilitators were reported such as practical, social/emotional, and financial help from their employer [16].

To our knowledge, the current study is the first to directly confront the opinions of cancer survivors with those of managers to identify managerial actions to be taken, at each stage of the RTW process, in order to promote the RTW of an employed cancer survivor. Implementing the actions “listen”, “communicate”, and “tailor” right from the disclosure phase, will allow effective collaboration between the manager and the cancer survivor, which is a pre-requisite for a successful RTW by actively engaging the cancer survivor in problem solving [20, 36]. “Referring to internal reintegration programs” is also important as it indicates that all the actions identified should not be the sole responsibility of the manager, as identified in the collective consultation phase, rather that the actions should be distributed to different managerial stakeholders within the company if possible (e.g., line manager, HR manager, employer) [20, 37]. If the actions are implemented by a single person, that individual could experience significant mental burden and paradoxical injunctions (i.e., supporting the RTW of the cancer survivor while maintaining a high level of productivity) [20]. In addition, the experts identified a certain hierarchic system in the managerial actions. Some should be regarded as overarching actions, such as “listen”, “tailor” and “collaborate”. Successful implementation of these managerial actions could lay a pre-requisite for a successful RTW support by the manager in the implementation of more practical managerial actions, such as “offer re-integration programs” and “support practically” [38]. It is therefore advisable to not regard the managerial actions as distinct actions to be taken independently of each other and solely by the line manager. Rather, the actions should be considered as an interplay of managerial actions, which are a shared responsibility of all stakeholders on the employer’s side [10, 21].

The main difference between the managerial actions that were selected in the current study compared to the study by Greidanus et al. [10] concerns privacy-related actions (i.e., “respect privacy” and “seek balance between privacy and support”). The experts considered privacy-related actions to be important for all RTW phases in the French context, while a previous study has shown that managers and cancer survivors did not prioritize these actions for any of the RTW phases in the Dutch context [10]. During the collective production phase of the current study, the elements discussed related to the private life mainly concerned the limits of support provided by the manager without being intrusive. Since salary compensation in France is covered by the social security system [39], it seems difficult for managers to draw the line between offering support actively (e.g., “support emotionally”, “show appreciation”) and distancing themselves from the cancer survivor who is undergoing treatment and who may really want to take some time off work, for example to fully recover. In the Netherlands, employers have far-reaching financial and practical responsibilities during the first two years of an employee’s sick leave [40]. So, cancer survivors expect them to take actions and therefore cancer survivors and employers are of the opinion that employer-related actions such as “providing support”, “allowing sufficient sick-leave” are prioritized over respecting the cancer survivor’s privacy [41].

Some managerial actions should also be implemented differently in France, when compared to other countries such as The Netherlands [39, 40]. For example, the provision of sick leave is done by French general practitioners. The managerial action “allow sufficient sick leave” thus includes for managers accepting and implementing the advice of the general practitioner correctly without making derogatory remarks [42]. The action “support financially” should also be implemented as helping with the paperwork, since financial support is organized through healthcare insurance. These differences, especially in terms of responsibilities, lead to a different involvement of managers in supporting cancer survivors’ RTW [21, 23]. Therefore, the legislative system of a specific country can serve as a brake or a lever for implementing managerial actions to support the RTW of cancer survivors [12, 21, 23, 38, 43].

The ARENA model of work disability prevention is an important avenue for explaining policy differences in managerial actions facilitating the RTW of cancer survivors [44]. Although these actions should primarily be implemented by the manager and, preferably, in agreement with the employed cancer survivor, their occurrence as well as their impact does not depend solely on the dyad. Actions such as “create a positive atmosphere” and “balance interests” (which both reached consensus on in all RTW phases) clearly show that the work team and the company policy must be considered in the process. Still in line with the ARENA model [44], such actions do not only depend on the dyad, but may also be influenced by different systems, such as the legislative and insurance system, the health care system, and the overall societal, and political context of a country. For example, de Rijk and colleagues found that these systems mainly shape the exact details of the RTW support while there was also a lot of consistent needs of employers across countries [21]. Considering these differences, it is advisable to always regard the important managerial actions to be taken during the RTW of cancer survivors in light of the respective country’s legal context [21]. Therefore, the actions should be seen in a holistic way to better understand the different levels of interventions and the levers to activate, to facilitate both the RTW of a cancer survivor and the effective implementation of these actions by the manager.

Differences between the study of Greidanus et al. [10] and the current study fall mainly into three methodological aspects. Firstly, the method used to reach consensus differed (i.e., TRIAGE method versus Delphi method) [10, 25–27]. In the case of the Delphi method, the consensus is built on the basis of several individual representations of the actions to be taken by managers to support the RTW of cancer survivors [10, 25]. This method comes with more statistical power, as the recommended number of experts exceeds those of the TRIAGE method [25–27]. In addition, the burden for participation is lower than for the TRIAGE method, due to the absence of an extensive collective consultation. On the other hand, the TRIAGE method is more dynamic, as it includes a discussion phase when divergences emerge between experts [26, 27]. During this phase, the individual experts can outline the context of their thoughts to each other, allowing them to make a well-considered and final decision on the importance of a certain managerial action. As a result, the consensus is built on the basis of a shared representation of the actions to be taken by managers to support the RTW of cancer survivors [26, 27]. Secondly, all experts (i.e., cancer survivors and managers) in the Greidanus et al. study were asked to select the ten most important managerial actions per RTW phase [10]. Since the selection of important managerial actions was limited to ten, the experts had to prioritize them, resulting in less managerial actions that reached consensus (i.e., ≥ 80% threshold) within an expert group. In the current study, more actions reached consensus, since the only limit was that the actions selected for collective consultation had to reach at least the 15th place of that specific RTW phase during the individual consultation. Thirdly, Greidanus et al. [10] analyzed the perspectives of managers and cancer survivors separately to identify potential differences between these perspectives. Conversely, the current study analyzed the perspectives of managers and cancer survivors jointly, as the aim was to reach consensus between the two parties.

### Limitations and Strengths

The main limitation of this study is the high dropout rate between the individual consultation phase and the collective consultation phase due to medical reasons, professional duties, or technical issues. Since the results are based on numbers and percentages, the sample size could be considered too small. However, the recommended group size of six to twelve for the TRIAGE method was still followed in the collective consultation phase (i.e., eight experts) [26, 27], and the guidelines of Ayre & Scally were carefully respected [33]. In addition, the collective consultation contained an extensive discussion of five hours, with all experts having ample possibilities to share their thoughts, resulting in a rich and useful collective consultation. On the other hand, the lengthy collective consultation could also be regarded as a limitation since it was rather a long time for the experts to stay focused. The five hours were needed to resolve some technical problems, introduce every expert, remind experts of the objectives of the study, and finally, discuss all managerial actions thoroughly. Several breaks were included to ensure that all experts stayed focused until the end of the session. Moreover, we did not identify the type of work of the experts that would have allowed us to have a better understanding of their work environment, and in turn to interpret the results in the light of their occupational context. Moreover, managers in the expert group mostly worked at large-sized companies. It is known that the work environment impacts RTW experiences and trajectories [16], and specific challenges are faced in smaller companies (e.g., limited possibilities for work adjustments and less access to supporting services) [45, 46]. Therefore, the generalizability of the managerial actions, that reached consensus in this study, to smaller organizations is yet unclear.

The main strength of this study lies in the possibility for discussion during the collective consultation, which allowed the heterogeneous group of experts (i.e., managers with various positions and cancer survivors) to understand each other’s context in the complex process of returning to work after cancer. This collective consultation thus allowed a shared representation of this process between the experts, which resulted in consensual thinking concerning the actions to be taken into account [26, 27]. The presence of cancer survivors who were also managers facilitated this process. The current study also has the strength to present a certain timeframe (i.e., Disclosure, Treatment, RTW plan, Actual RTW) for important managerial actions. Although some managerial actions are important for all RTW phases, several actions were found to be specific for only one or a few phases. The results complement those of Greidanus et al. [10] by identifying all the actions, not just the most important ones, to be carried out by managers to facilitate the RTW of a cancer survivor. Another strength of the current study is that the cancer survivors in the expert group were all trained as expert-patients. Although these cancer survivors were on average diagnosed with cancer six years ago, each cancer survivor used their own experience with recent other RTW experiences of cancer survivors when participating in this study, enhancing the generalizability of the results.

### Implications for research

In this study, the managerial actions were identified to facilitate the RTW of cancer survivors. On the other hand, some cancer survivors who continue to work during treatment also require managerial actions to better support them in their job retention process. We assume that most managerial actions would fit the need of cancer survivors who continue to work during treatment (except for e.g., “allow sufficient sick leave”). However, based on the initial actions used in this study, future research could be proposed to identify managerial actions that promote job retention for individuals diagnosed with cancer. Furthermore, the TRIAGE method includes the collection of a multitude of qualitative data during the collective consultation (e.g., notes, recording of the discussion, visual support). These data may possibly provide more substance to the discussion. We encourage future research to identify a way to implement all the data collected throughout the TRIAGE steps.

### Implications for cancer survivors

Although this study focuses on the managerial actions, the cancer survivor remains at the heart of the process. Identification of managerial actions and their implementation allows cancer survivors to feel fully supported professionally and contribute to a sustainable RTW [10, 11, 34]. Furthermore, the additional action indicating that support should be tailored, suggests that the cancer survivor should be able to choose whom to be supported by within the company. Depending on the legal responsibilities within the company and the country and depending on the nature of the relationships of the cancer survivor, this person can be a manager, one or more colleagues, or the HR department.

## Conclusion

Managerial actions are of paramount importance in the process of returning to work after cancer. Applying the TRIAGE method, a consensus has been established between cancer survivors and managers to identify 25 important managerial actions distributed into each stage of the RTW process. These actions should be conducted collectively within the company, offering support adapted to the needs and desires of each cancer survivor returning to work. The identification of these actions will promote the emergence of assessment and training tools for managers to better support the RTW of cancer survivors.

## Electronic supplementary material

Below is the link to the electronic supplementary material.

Supplementary Material 1

## Data Availability

The datasets used and/or analyzed during the current study are available from the corresponding author on reasonable request.

## List of abbreviations

AF3M: French Association of Patients of Multiple Myeloma (French:Association Française des Malades du Myélome Multiple).
HR: Human resources
ReWork-QoL: Quality Of Life And Sustainable Return to Work.
RTW: Return to work.
SD: Standard deviation.
SIRIC: Integrated Cancer Research Site (French:Site de Recherche Intégrée sur le Cancer) - Imaging and Longitudinal
ILIAD: Investigations to Ameliorate Decision-making.
TRIAGE: Technique for Research of Information by Animation of a Group of Experts.
