# Supplementary material for: Identification of actions to be taken by managers to facilitate the return to work of cancer survivors: Consensus between managers and cancer survivors
Source: BMC Public Health. 2022 Oct 12;22:1905. doi: 10.1186/s12889-022-14271-w (PMC9555691; doi:10.1186/s12889-022-14271-w)
Supplement: Supplementary file 1 — Supplementary Material 1 [file 12889_2022_14271_MOESM1_ESM.docx]

**Supplementary material**

**Table S 1.** Number of experts who considered the action important in the collective consultation phase (N = 8)

| **Actions** | **Phase 1: Disclosure**  **n** | **Phase 2:**  **Treatment**  **n** | **Phase 3: Return to work plan**  **n** | **Phase 4: Actual return to work**  **n** |
| --- | --- | --- | --- | --- |
| 1. **Support practically** – Provide the employee with cancer with practical support (e.g., adapting tasks, workplace and working hours) | 4 | - | X | X |
| 2. **Assess work ability** – Assess the extent to which the employee with cancer is able to work in the right manner | 0 | - | X | X |
| 3. **Show appreciation** – Give the employee with cancer the feeling that you want them back at work | X | X | X | X |
| 4. **Communicate** – Communicate effectively with the employee with cancer (in terms of tone, intensity, subjects and channels) | **7** | X | X | X |
| 5. **Support emotionally** – Support the employee with cancer emotionally (e.g., showing interest, being involved and understanding) | **7** | **8** | X | **8** |
| 6. **Adjust expectations** – Adjust expectations regarding the performance of employee with cancer to their current situation | 3 | - | - | X |
| 7. **Allow sufficient sick leave** – Allow sufficient sick leave and not putting pressure on the employee with cancer to return to work | **8** | X | X | - |
| 8. **Treat normally** – Treat the employee with cancer as if they are not ill (e.g., avoid inappropriate treatment, including being too protective or concerned) | **7** | - | - | **7** |
| 9. **Plan return to work** – Make a plan for the employee’s return to work in consultation with them | - | 0 | X | **8** |
| 10. **Handle unpredictability** – Try to cope as well as possible with the unpredictability of the illness and the absence of the employee with cancer | **8** | X | X | **8** |
| 11. **Reduce work pressure** – Reduce the pressure of work on the employee with cancer | 5 | 6 | - | X |
| 12. **Radiate a positive attitude** – Radiate a positive attitude when guiding the employee with cancer | X | **8** | X | **8** |
| 13. **Respect privacy** – Respect the privacy of the employee with cancer | X | X | X | **8** |
| 14. **Deal with colleagues** – Inform and supervise colleagues of the employee with cancer | - | **8** | X | **8** |
| 15. **Collaborate** – Collaborate with the employee with cancer | - | - | - | **8** |
| 16. **Create a positive work atmosphere** – Create a positive atmosphere at work, whether or not the employee with cancer is present | **8** | **8** | - | X |
| 17. **Offer reintegration programs** – Offer the employee with cancer external reintegration programs (e.g., third-party support services of fitness programs) | - | 1 | X | - |
| 18. **Balance interests** – Try to cope as well as possible with the different interests at stake (e.g., those of the company, the employee with cancer and their colleagues) | **8** | X | X | X |
| 19. **Provide time for reorientation and retraining** – Provide the employee with cancer with time for reorientation and retraining | - | - | X | 6 |
| 20. **Seek balance between privacy and support** – Seek the right balance between respecting the privacy of the employee with cancer and offering them support | X | X | X | X |
| 21. **Support financially** – Support the employee with cancer financially (e.g., continue to pay them during sick leave or help them with benefits applications) | **8** | **7** | - | - |
| 22. **Comply with legislation** – Comply strictly with the obligations imposed by the law | - | - | - | - |
| 23. **Search for external support for yourself (manager)** – Seek out external support for yourself as the employer of an employee with cancer (e.g., from the occupational physician, other employers or a psychologist). *Note that this external support does not target the employee.* | - | **8** | - | - |
| 24. **Possess or seek knowledge of cancer** – Possess or seek out general knowledge of cancer, its treatment and its possible consequences for work | - | - | - | - |
| **Additional action 1 – Listening:** Provide active listening to the difficulties and needs expressed by the employee diagnosed with cancer. | **8** | **8** | **8** | **8** |
| **Additional action 2 – Refer to internal reintegration programs:** Refer to the relevant experts within the company (e.g., occupational physician, social worker, human resources services, tutors, experience experts) | **8** | **8** | **8** | **8** |
| **Additional action 3 – Tailoring:** Tailor the support to the needs of the employee cancer | **8** | **8** | **8** | **8** |
| *Notes.* The numbers presented correspond to the number of experts who responded “Yes” for each action in each phase  - : actions that were not discussed in the collective consultation  X: actions on which the expert already reached consensus during the individual consultation  **In bold:** actions on which the expert reached consensus during the collective consultation | | | | |
